# Free-Living Hip Accelerometry Detects and Forecasts Frailty Decline in a Sample of Community-Dwelling Older Adults

**DOI:** 10.1093/geroni/igaf122.1766

**Published:** 2025-12-31

**Authors:** Megan Huisingh-Scheetz, Benjamin Kramer, Yanan Long, Michelangelo Pagan, Sylvia Brown, Andrey Rzhetsky

**Affiliations:** The University of Chicago, Chicago, Illinois, United States; Case Western Reserve University, Cleveland, Ohio, United States; The University of Chicago, Chicago, Illinois, United States; The University of Chicago, Chicago, Illinois, United States; National Opinion Research Center at The University of Chicago, Chicago, Illinois, United States; The University of Chicago, Chicago, Illinois, United States

## Abstract

Frailty is a geriatric syndrome combining reduced strength, exhaustion, slow gait, weight loss, and low activity, foreboding adverse health outcomes. Early detection of frailty is critical for mitigating risk, but the standard frailty detection is time-consuming. Non-invasive, wearable accelerometers capture physical activity and sleep patterns and may detect frailty changes. In this retrospective study, 7-days of free-living data from hip accelerometers were used to both detect concurrent frailty phenotype (range 0-5, nonfrail=0 vs pre-frail or frail=1-5 points) and forecast frailty increase (1+ point worse frailty score vs no change or improved score) over 1 year in community-dwelling older adults. We extracted 115 accelerometry features from the hip accelerometry readings (activity, sleep, harmonic variables) using open-source code. Five machine learning models included all accelerometry features plus demographic and health characteristics. The models were trained on 85% of the sample and were tested on 15% of the sample. In this presentation, we will share the best-performing cross-sectional model (F1=0.84, AUROC=0.84) and the best performing longitudinal model (F1=0.87, AUROC=0.84). These findings suggest that wearable sensors’ data may be useful for real-time frailty screening and monitoring. Integrating accelerometry into clinical care could facilitate earlier detection of at-risk individuals and improve outcomes.

